# Splenectomy in Patients with Sickle Cell Disease in Tabuk

**DOI:** 10.3889/oamjms.2016.034

**Published:** 2016-02-29

**Authors:** Asmaa Ghmaird, Mohammad Mohammad Alnoaiji, Sawsan Al-Blewi, Shaimaa Zaki, Ahmad El-lewi, Nehal Ahmad

**Affiliations:** 1 *University of Tabuk, Medicine Faculty, Tabuk, Saudi Arabia*; 2 *North West Armed Forces Hospital, Surgery Department, Tabuk, Saudi Arabia*; 3 *University of Tabuk, Pediatric Department, Tabuk, Saudi Arabia*; 4 *Medical Health Division National Research Center, Child Health Department, Cairo, Egypt*; 5 *North West Armed Forces Hospital, Tabuk, Saudi Arabia*

**Keywords:** sickle cell disease, splenectomy, pediatrics, spleen sequestration, acute chest syndrome, blood transfusion

## Abstract

**BACKGROUND:**

Sickle cell disease is a common genetic disease in Saudi Arabia; it is an autosomal recessive disorder characterized by production of abnormal hemoglobin S and is associated with high morbidity and mortality. Acute splenic sequestration is a life-threatening complication for this disease. Prophylactic splenectomy is the only effective strategy for preventing future life-threatening episodes.

**AIM:**

The aim of this study was to study hospital records for all children aged 2 to 12 year old with Sickle cell disease who underwent splenectomy in Tabuk in Saudi Arabia.

**METHODS:**

Records of 24 children (13 males, 11 females) who underwent splenectomy in surgery department of King Salman North West Armed Hospital, Tabuk, Saudi Arabia between 2008 and 2015 were reviewed retrospectively and analyzed for age, sex, indications for splenectomy, surgical technique, preoperative and postoperative length of stay, operative and postoperative complications, acute chest syndrome, painful crises, blood transfusion and fever (preoperative and postoperative).

**RESULTS:**

We stressed on the information about the details of operation, the frequency of blood transfusion, fever, acute chest syndrome and painful crisis before and after operation.

**CONCLUSION:**

Here we found that blood transfusion frequency decreased after splenectomy.

## Introduction

Sickle cell disease (SCD) is an autosomal recessive disorder characterized by production of abnormal hemoglobin S and is associated with high morbidity and mortality. It is relatively common in Saudi Arabia as consanguineous marriage rates exceed 50% [1]. The reported prevalence for sickle-cell trait ranges from 2% to 27%, while up to 2.6% has SCD [2]. The spleen is one of the most common and early organs to be involved in SCD. It is commonly enlarged during the first decade of life then it undergoes progressive atrophy because of repeated attacks of vasoocclusion and infarction and these cause autosplenectomy. Sometimes splenomegaly persists into older age group or even into adulthood. Necessary splenectomy is done for a variety of reasons including acute splenic sequestration crisis, hypersplenism, massive splenic infarction and splenic abscess [3]. Splenic complications of SCD are associated with an increased morbidity and sometimes it may lead to mortality. To obviate this, There is a paucity of evidence to support that splenectomy, by whatever means, should be performed to improve survival and decrease morbidity [4]. Acute splenic sequestration is a life- threatening complication [5]. It is considered the second leading cause of death after infection in the first decade of life in patients with SCD. It has high mortality rates and in survivors there will be frequent recurrence of first attack [4]. Repeated episodes of splenic sequestration are common, happening in half of patients, especially within 6 months of the previous episode [6]. The only effective strategy for preventing future life-threatening episodes is the Prophylactic splenectomy which performed after an acute episode has resolved. Splenectomy may be performed with either celiotomy (open Splenectomy), or with minimal access approach (laparoscopic Splenectomy) [6, 7].

The aim of this study was to study hospital records for all children aged 2 to 12 year old with Sickle cell disease who underwent splenectomy in Tabuk in Saudi Arabia.

## Materials and Methods

Records of 24 children (13 males, 11 females) who underwent splenectomy in surgery department of King Salman North West Armed Hospital, Tabuk, Saudi Arabia between 2008 and 2015 were reviewed retrospectively and analyzed for age, sex, indications for splenectomy, surgical technique, preoperative and postoperative length of stay, operative and postoperative complications, acute chest syndrome, painful crises, blood transfusion and fever (preoperative and postoperative). The patients’ demographics are shown in Table 1.

**Table 1:** The patient’s demographics

Age (mean ± standard deviation)	10 ± 4 (4 – 16)

Gender	

Male	13 (54.2)

Female	11 (45.8)

Preoperative diagnosis and indications for splenectomy were established in pediatric and hematology departments. All patients were prepared in surgery department for splenectomy and they were followed there after the procedure. Indications for surgery were spleen sequestration episode (once or multiple), hypersplenism, and symptomatic splenomegaly.

All patients received preoperative vaccination with polyvalent pneumococcal, meningococcal, and Haemophilus influenza vaccines before two weeks at least Patients were evaluated with ultrasonography (US) to determine the size of spleen, to rule out presence of concomitant gallstones, and to determine the presence of accessory spleens. The hematology policy in this center is: all patients with Hb level of less than 10 g/dL on admission were transfused preoperatively with packed erythrocytes to increase their Hb level to 10.

Routine antibiotic prophylaxis was started at the onset of surgery and continued for 3 days thereafter. Antibiotics of choice were a third-generation cephalosporin. Operation was performed under general anesthesia, all operation were complete splenectomy, 13 were opened (54.2%), and 8 were Laparoscopic (33.3%), while 3 were Laparoscopic turned to open.

## Results

From 2008 to 2015, the records of 24 patients with SCD had splenectomy in North West armed forces hospital. Their age ranging from 4 to 16 years mean age was 10 years ± 4. Gender was 13 males (54.2%), 11 females (45.8%). Some data from records was missed, 10 patients 41.7% had Hb SS, 13 patients 54.2% Had Hb Sβ thalathemia, 1 patient with missed HB electrophoresis.

**Table 2:** The technique details of operation

	Variable	Frequency	Percent
Type of procedure	Complete	24	100.0

Technique of procedure	Laparoscopic	8	33.3

	Open	13	54.2

	Laparoscopic turn to open	3	12.5

Concurrent procedures	None	17	70.8

	Cholycystectomy	7	29.2

Indications of splenectomy were; two patients with single spleen sequestration episode (8.3%), 13 patients with multiple spleen sequestration episode (54.2%), three patients with hypersplenism (12.5%), three patients with multiple spleen sequestration episode & hypersplenism (12.5%), one patient with single spleen sequestration episode & hypersplenism (4.2%). The indications for two operations were missed The age at operation ranged from one year six month old to twelve years old with Median age 4 with 10 patients aged 3 years old and less and nine patients aged 6 years old and more.

**Table 3:** Hb electrophoresis

Variable	Median	Inter quartile range	

		Percentile 25	Percentile 75
Hb electrophoresis A1	.00	.00	2.60

Hb electrophoresis A2	3	2	3

HB electrophoresis S	70.70	61.00	79.40

Hb electrophoresis F	20.30	16.10	29.00

Hundred percentages of the patients underwent complete splenectomy. Thirteen patient underwent open splenectomy representing 54.2% of the patients, eight patents underwent laparoscopic splenectomy representing 33.3% while 3 patients had laparoscopic splenectomy that turned to open. Seven patients had cholycystectomy together with splenectomy. Twenty one patient needed blood transfusion during the operation (87.5%) and 3 patients needed no blood transfusion.

After the operation the median hospital stay was 4 days ± 1 compared to 2 days ± 1 preoperative. The patients Hemoglobin before the operation was 9 ± 1 gm/dl (before blood transfusion), while after the operation the median was 11 ± 2. The median platelet count preoperative was 278*103 ± 118, the number increased to 363*103 ± 391 post-operatives. The frequency of ICU admission from acute chest syndrome was only one patient (4.2%) preoperative while the frequency of ICU admission from acute chest syndrome was 6 (25%) post- operative. Wilcoxon signed-rank test was used for comparison between pre and postoperative variables.

**Figure 1:**
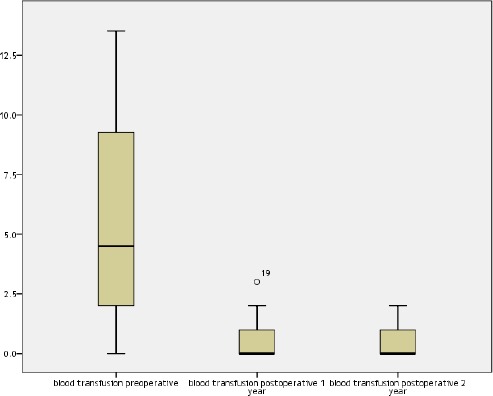
*Blood transfusion pre and ostoperative*.

Blood transfusion pre and postoperative are shown in Figure 1. The median rate of blood transfusion per year preoperative is 4.5 with the 25th quartile 2.0 and the 75th quartile 9.6, while median rate of blood transfusion for 1st year postoperative and 2nd year post- operative is 0, the 25th quartile is 0 and the 75th is 1 (P value < 0.001).

Regarding fever, preoperative the median number of fever episodes preoperative is 0.75, the 25th quartile is 0 and the 75th quartile is 2. One year postoperative the median increases to 1, the 25th quartile remains 0 and the 75th quartile is 1. 2nd year post- operative the median drops to 0, the 25th quartile is 0 and the 75th quartile is 1 (P = 0.382).

**Figure 2:**
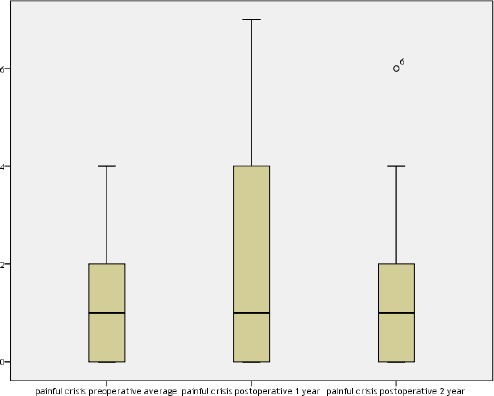
*Painful crisis pre and postoperative*.

The episodes of painful crisis preoperative median is 1, the 25^th^ quartile is 0 and the 75th quartile is 2, one year postoperative the median remains one, the 25th quartile is 0 and the 75th quartile is 4.25, the 2nd year postoperative the median remained 1, the 25th quartile is 0 and the 75th quartile is 2.25 (P value = 0.479). Results are shown in Figure 2.

Fridman test for repeated measures as in comparison between variables pre, 1 year postoperative, and 2 year postoperative.

## Discussion

Sickle cell disease (SCD) is a generic term referring to a group of disorders characterized by red blood cell deformity. Sickle cell disease is characterized by recurrent vasoocclusive episodes associated with accelerated hemolysis [8].

Splenectomy is one of the most common procedures required for patients with SCD [9]. The major indications for splenectomy in sickle cell children include splenic sequestration episodes and severe hemolysis secondary to suspected hypersplenism [3, 9-12]. Nowadays with the advances of minimally invasive surgery, LS is believed by many to be a gold standard [9, 13].

Acute chest syndrome is an important cause of mortality and morbidity in children with SCD [14] (21). Acute chest syndrome is characterized by basal pulmonary infiltrates that always involve the lung on the side of the surgery and either remains localized to that lung or progresses to involve both lungs [15].

In our study the indication of spleenectomy in most cases was recurrent splenic sequestration episodes and to a lesser extent hypersplenism or both, other studies in addition to Recurrent acute splenic sequestration crisis and hypersplenism found also splenic abscess, splenomegaly with a nonfunctioning spleen and massive splenic infarction to be less common indications [3].

In our study 100% of the patients underwent complete spleenectomy. 54.2% of the patients underwent open spleenectomy, while 33.3% of the patients underwent laparoscopic spleenectomy 12.5% had laparoscopic spleenectomy that turned to open. In other studies The ratio of laparoscopic vs open splenectomy was approximately 3:2 [8]. Since the first report of laparoscopic splenectomy in 1993 [1], the procedure has gained widespread acceptance and is considered safe and effective [16]. Seven patients (29.2%) had cholycystectomy together with spleenectomy. Twenty one patient needed blood transfusuion during the operation (87.5%) and 3 patients needed no blood transfusion. There was no mortality and post operative complications were ileus in one patient and fever with diarrhea in another patient (8.4%), in other studies Twenty-eight (21%) of our the patients had splenectomy and cholecystectomy There was no mortality, but 8 (6%) developed postoperative complications [8].

In our study the median hospital stay after the operation was 4 days ± 1 compared to 2 days ± 1 preoperative. Other studies have found the mean length of postoperative stay for laparoscopic procedures to be 3 days, which is similar to [3, 11, 13]. Laparoscopic splenectomy has been shown to confer a clear benefit of shorter hospital stay when compared to open splenectomy in sickle cell children [17].

In the current study the patients Hemoglobin before the operation were 9 ± 1 gm/dl, while after the operation the median was 11 ± 2. The median platelet count preoperative was 278 ± 118, the number increased to 363 ± 391 post operative.

The median rate of blood transfusion per year preoperative was 4.5 with the 25th quartile 2.0 and the 75th quartile 9.6, while median rate of blood transfusion for 1st year postoperative and 2nd year post operative is 0, the 25th quartile is 0 and the 75th is 1 (P value <.001).

Lesher and collegues have found in a previous study there was a 38% decrease in the number of units transfused during 0 to 6 months postsplenectomy and a 45% decrease during 6 to 12 months postsplenectomy [8]. Despite a decrease in the number of units transfused postsplenectomy, all hematologic parameters remained stable or improved. Hematocrit increased marginally and reticulocytes decreased, indicating a decreased red cell turnover after the splenectomy. These results taken together indicate that there was an improvement in the survival of the transfused red cells [8]. Another study showed that the postoperative hematocrit and reticulocytes significantly improved in children in Saudi Arabia who underwent splenectomy for hypersplenism [3]. Svarch et al [6] showed significant improvement in hemoglobin concentration (6.0 vs. 7.7; P = .01) with decrease in transfusion after partial splenectomy for acute splenic sequestration with concurrent decrease in transfusion requirement [18].

In our study The frequency of ICU admission from acute chest syndrome was only one patient (4.2%) preoperative while the frequency of ICU admission from acute chest syndrome was 6 (25%) post operative.in previous studies the rate of acute chest syndrome was 5 to 15% [16, 19].

## References

[1] Meyer BF (2005). Strategies for the prevention of hereditary diseases in a highly consanguineous population. Annals of Human Biology.

[2] Jastaniah W (2011). Epidemiology of sickle cell disease in Saudi Arabia Ann. Saudi Med.

[3] Al-Salem AH (2006). Indications and complications of splenectomy for children with sickle cell disease. J Pediatr Surg.

[4] Owusu-Ofori S, Hirst C (2013). Splenectomy versus conservative management for acute sequestration crises in people with sickle cell disease. Cochrane database Syst Rev.

[5] Kliegman R, Stanton B, St. Geme J, Schor N (2015).

[6] Ziegler M, Azizkhan RG, von Allmen D, Weber T (2014). Operative Pediatric Surgery. McGraw Hill Professional.

[7] Coran A, Scoot Adzick N, Krummel T, Laberger J, Shamberger R, Caldamone A (2012). Pediatric surgery.

[8] Lesher AP, Kalpatthi R, Glenn JB, Jackson SM, Hebra A (2009). Outcome of splenectomy in children younger than 4 years with sickle cell disease. Journal of Pediatric Surgery.

[9] Buck J, Davies SC (2005). Surgery in sickle cell disease. Hematol Oncol Clin North Am.

[10] Holterman AX, Adams KN, Seeler RA (2006). Surgical management of pediatric hematologic disorders. Surg Clin North Am.

[11] Rescorla FJ, Grosfeld JL, O’Neill JA, Fonkalsrud EW (2006). The spleen. Pediatric surgery.

[12] Sounthararajah Y, Vichinsky E, Embury SH, Hoffman R, Benz EJ, Shattil SJ (2005). Sickle cell disease. Hoffman:hematology:basic principles and practice.

[13] Rescorla FJ, Breitfeld PP, West KW (1998). A case controlled comparison of open and laparoscopic splenectomy in children. Surgery.

[14] Vishinsky EP, Neumayr LD, Earles AN (2000). Causes and outcomes of acute chest syndrome in sickle cell disease. National Acute ChestSyndrome Study Group. N Engl J Med.

[15] Wales PW, Carver E, Crowford MW (2001). Acute chest syndrome after abdominal surgery in children with sickle cell disease:is a laparoscopic approach better?. J. Pediatr Surg.

[16] Murawski M, Patkowski D, Korlacki W (2008). Laparoscopic splenectomy in children—a multicenter experience. J Pediatr Surg.

[17] Goers T, Panepinto J, Debaun M (2008). Laparoscopic versus open abdominal surgery in children with sickle cell disease is associated with a shorter hospital stay. Pediatr Blood Cancer.

[18] Svarch E, Nordet I, Valdes J (2003). Partial splenectomy in children with sickle cell disease. Haematologica.

[19] Kokoska ER, West KW, Carney DE (2004). Risk factors for acute chest syndrome in children with sickle cell disease undergoing abdominal surgery. J Pediatr Surg.

